# PROTOCOL: Effectiveness of Sexual and Reproductive Health Blended Learning Approaches for Capacity Strengthening of Health Professionals in Low‐ and Middle‐Income Countries: A Systematic Review

**DOI:** 10.1002/cl2.70028

**Published:** 2025-03-11

**Authors:** Elizabeth A. Kumah, Florence Mgawadere, Alice Ladur, Zainab Suleiman, Yusupha Sanyang, Sarah A. White, Nicholas Furtado, Uzochukwu Egere, Charles Ameh

**Affiliations:** ^1^ Liverpool School of Tropical Medicine, International Public Health Liverpool UK; ^2^ The Global Fund Geneva Switzerland

**Keywords:** blended learning, capacity strengthening, health professionals, low‐ and middle‐income countries, sexual and reproductive health

## Abstract

This is the protocol for a Campbell systematic review. The objectives are as follows. The primary objective of this systematic review is to evaluate and synthesise both published and unpublished literature on the effectiveness of sexual and reproductive health blended learning approaches for capacity strengthening of healthcare practitioners in LMICs. Within this context, sexual and reproductive health interventions refer to any of the following four key interventions or services aimed at improving maternal and newborn health (Starrs et al. 2018): (a) antenatal, childbirth and postnatal care, including emergency obstetric and newborn care, (b) safe abortion services and treatment of the complications of unsafe abortion, (c) prevention and treatment of malaria, tuberculosis, HIV and other sexually transmitted infections in pregnant women and d) family planning. In this systematic review, blended learning is defined as any teaching and learning method that combines face‐to‐face learning with e‐learning or online learning. The component of face‐to‐face and online learning may include any of the components identified by Alammary (2019): (1) face‐to‐face instructor‐led, where students attend a class and an instructor presents teaching and learning materials, with little engagement from students; (2) face‐to‐face collaboration, where students work together in class, for example, in discussion groups; (3) online instructor‐led, where instruction is delivered online and facilitated by an instructor who sets the pace (e.g., virtual classrooms); (4) online collaboration, where students work together online with their peers, for example, online learning communities; and (5) online self‐paced, where students study at their own pace and time, and from their chosen location, for example, watching videos, online reading. Specifically, this systematic review will answer the following research questions: (1) What sexual and reproductive health blended learning approaches have been used in LMICs? (2) Does participating in sexual and reproductive health blended learning interventions alone (i.e., compared with no intervention) improve the effective provision of care among healthcare workers in LMICs? (3) Does participating in sexual and reproductive health blended learning interventions compared with non‐blended learning approaches (such as conventional face‐to‐face learning or pure e‐learning) facilitate the effective provision of care among healthcare workers in LMICs (measured by, e.g., self‐reports of effective maternal and neonatal care)? (4) What is the cost‐effectiveness of sexual and reproductive health blended learning compared with non‐blended learning approaches (i.e., face‐to‐face learning or e‐learning)? (5) What factors affect the effectiveness of sexual and reproductive health blended learning interventions (e.g., characteristics of participants, type of intervention, course content, setting and mode of delivery)? (6) Do sexual and reproductive health blended learning interventions targeted at healthcare practitioners working in LMICs lead to improvement in patient outcomes (e.g., reduced maternal and neonatal mortality, patient satisfaction reports)?

## 1 Background

### 1.1 Description of the Condition

Universal access to adequate sexual and reproductive health care is a basic human right (UN 2015) and a key element of the universal health coverage agenda established as part of the United Nations' sustainable development goals. Good sexual and reproductive health refers to a state of complete mental, physical and social wellbeing in all matters concerning an individual's reproductive system and sexuality. It includes the right to a safe and satisfying sex life, as well as the ability to make an informed decision regarding one's sexual and reproductive health (UNFPA 2016). Access to accurate sexual and reproductive health information is essential to empower individuals to be involved in decisions regarding their reproductive health, reduce unplanned pregnancies and abortions, and support healthy pregnancies, safe deliveries and ultimately healthy babies, in women who decide to have children (WHO 2019a).

Sexual and reproductive health inequalities exist, and these vary according to gender, socioeconomic status, education level, ethnicity and availability of resources (Hall et al. 2012). Evidence indicates that the burden of sexual and reproductive ill‐health is relatively high in women, and may result from unplanned pregnancies, high maternal, neonatal and child mortality and stillbirths (Arregoces et al. 2015). Moreover, individuals in low‐ and middle‐income countries (LMICs) face difficulties accessing appropriate reproductive health services due to limited resources (Naal et al. 2020). As such, despite considerable progress in improving reproductive health services, there is still a disproportionately high level of morbidity and mortality among women of childbearing age in LMICs compared to those of high‐income countries (Mariani et al. 2017). The World Health Organisation (WHO) estimates that in 2017 alone, almost 295,000 women died globally during or after pregnancy and delivery, and 94% of these deaths occurred in LMICs (WHO 2019b). This reflects a maternal mortality ratio of 462 per 100,000 live births in LMICs compared with 11 per 100,000 live births in high‐income countries. The high rates of maternal mortality in LMICs closely correlate with neonatal mortality figures in these countries. This is because when mothers receive inadequate antenatal, delivery and postnatal care services, their new‐borns are exposed to higher risks of morbidity and premature deaths (WHO 2020). Thus, it is imperative for all women to have high‐quality health care across the continuum of care.

Achieving improvement in maternal and neonatal health outcomes is a global health priority, and a key target for the sustainable development goal number three, targets 3.1, 3.2, 3.7 and 3.8, which are, reduction of global maternal mortality ratio to less than 70 maternal deaths per 100,000 live births; end to avoidable deaths of neonates and children under 5 years old; enhancing access to sexual and reproductive health services and achieving universal health coverage by 2030, respectively (UN 2015). To achieve these targets, timely management and treatment of pregnancy and delivery‐related complications are essential. All antenatal, delivery and postnatal care must be attended by skilled healthcare practitioners (Say et al. 2014). Yet, LMICs experience significant crises with health care, and concerns regarding the readiness and quality of the healthcare workforce have been raised (Nicol et al. 2019). Hence, there is an urgent need for training for healthcare practitioners to equip them with the knowledge and skills needed to deliver quality healthcare services.

Over the past three decades, international and national bodies have implemented capacity strengthening interventions (e.g., through training healthcare practitioners) to strengthen health systems and respond to the needs of service users (WHO 2019b). Several methods have been used to train healthcare practitioners, including face‐to‐face, electronic(e)‐learning, and a combination of a variety of methods (e.g., face‐to‐face training and e‐learning), termed blended learning (Frehywot et al. 2013).

An initial scoping of the literature revealed a plethora of research studies examining the various approaches that have been used to train healthcare practitioners and their effectiveness for capacity strengthening of practitioners in LMICs. Examples include Rosenberg et al. (2022); Millimouno et al. (2021); Bertman et al. (2019); Balasubramaniam et al. (2018); Yigzaw et al. (2019); Limaye et al. (2018); and Henschke et al. (2017). Capacity strengthening initiatives in healthcare refer to activities aimed at developing the abilities of healthcare practitioners and institutions to manage healthcare‐related issues (Kislov et al. 2014; Paul 1995). These initiatives have been recommended by DeCorby‐Watson et al. (2018) and Beran et al. (2017) as among the most effective approaches to respond to the healthcare challenges in LMICs, as they have the potential to improve the knowledge, skills and overall competence of healthcare practitioners.

In recent years, blended learning interventions for healthcare practitioners have gained momentum as a valuable initiative for strengthening the capacity of healthcare professionals, particularly in low‐resource settings (Al‐Shorbaji et al. 2015; Naal et al. 2020; WHO 2016). The use of blended learning and other innovative methods of teaching, such as pure e‐learning, has become even more necessary with the emergence of the global Coronavirus‐19 pandemic (Mpungose 2020). A cost‐effectiveness analysis of blended learning versus traditional face‐to‐face learning conducted by Maloney et al. (2015) revealed that a blended learning approach was more cost‐effective and led to improvement in the competencies of learners.

While there have been several published systematic reviews assessing the effectiveness of blended learning approaches, most of them were focussed on students, including pharmacy, medical, nursing and a combination of medical, nursing and allied health students (Balakrishnan et al. 2021; McCutcheon et al. 2014; Vallée et al. 2020; Rowe et al. 2012). No published systematic review exists that specifically assesses the effectiveness of sexual and reproductive health blended learning approaches aimed at strengthening the capacity of healthcare practitioners in LMICs. The current systematic review will identify and synthesise studies that have evaluated the effectiveness of blended learning approaches used in delivering sexual and reproductive health training packages to healthcare practitioners working in LMICs.

### 1.2 Description of the Intervention

Recent advancements in technology have not only transformed social lives but have led to significant changes in education (Garrison 2011), particularly, in relation to modes of delivery of educational programmes. Increasingly, learners have come to expect educational programmes to be delivered in a way that offers convenience and improved usability (Palfrey and Gasser 2013). These changes have been embraced in healthcare education and are considered beneficial, especially among healthcare practitioners working in remote and rural settings (Maloney et al. 2013; Wellard and Bethune 2000), where there are usually limited infrastructural and human resources. Indeed, some healthcare professionals seeking continuous professional development opportunities experience difficulties due to limited access to face‐to‐face education (Lenthall et al. 2011), resulting from geographical isolation or the lack of time to attend face‐to‐face sessions (Doorenbos et al. 2011). As such, alternative methods to traditional face‐to‐face education (such as pure e‐learning or blended learning, which is a combination of face‐to‐face and e‐learning) have been recommended as effective in overcoming these challenges. These alternate methods of education are even more important now, as there is an urgent need to strengthen the capacity of healthcare professionals in resource‐constrained settings (Naal et al. 2020).

E‐learning gained momentum in the mid‐1990s as the internet began to gain popularity (Garrison 2011). It can be defined as the delivery of education using the internet and other digital technologies, such as computers, CD‐ROMS, smartphones and DVD, and can be delivered both inside and outside the classroom (Clark and Mayer 2011; Frehywot et al. 2013). E‐learning is thought to be an efficient way of educating people as it transcends time, space and geographical boundaries (Moreira et al. 2015). The term ‘e‐learning’ has been used synonymously with terms, such as ‘online learning’, ‘internet‐based learning’, ‘web‐based learning’, distributed learning and ‘computer‐assisted learning’. While pure e‐learning and completely online learning have mostly been used synonymously, some authors have identified significant differences, which is in relation to the delivery platform. Pure e‐learning can take place without internet access, for example, using DVDs or CD‐ROMS to deliver educational content. However, completely online learning requires internet access and relies on a web‐based delivery platform (Frehywot et al. 2013). In this systematic review, the term e‐learning refers to the delivery of educational contents using computers and other digital technologies, with or without internet and includes both pure e‐learning and fully online learning.

E‐learning contents can be delivered in either synchronous or asynchronous formats (Kinshuk and Chen 2006). Synchronous delivery of educational contents refers to a tutor‐led, real‐time education where all learners are taught at the same time and can interact among themselves, through a virtual classroom platform. In asynchronous delivery, however, educational contents are transmitted and received at different time points, and can include pre‐recorded lectures, podcasts or simulation (Ruiz et al. 2006). It enables learners to participate in educational activities at any time and from any geographical location (Ruggeri et al. 2013). Communication methods such as emails, wikis, weblogs and online bulletin boards may be used in asynchronous educational content delivery.

Notwithstanding its benefits, e‐learning has potential limitations, including the feeling of isolation among learners in a virtual environment, and the high cost of developing and maintaining online platforms as well as preparing online materials (Wu et al. 2010). Moreover, the lack of face‐to‐face interaction among learners, problems associated with internet connectivity and poor instructional design have made e‐learning less appealing (Cook 2007). To overcome these limitations, blended learning has been recommended as a favourable alternative approach for healthcare education because it combines the advantages of both e‐learning and conventional face‐to‐face learning.

This systematic review will consider primary studies that assess sexual and reproductive health blended learning approaches targeted at healthcare practitioners working in low‐ and middle‐income countries. In this systematic review, sexual and reproductive health blended learning approaches/interventions refer to educational programmes in sexual and reproductive health that are aimed at strengthening the capacity of healthcare practitioners through a combination of traditional face‐to‐face learning, and e‐learning approaches (this may include self‐directed learning). We will consider for inclusion any type of educational programme that is delivered either as short or long certificate courses, diploma or degree courses, or in‐service training of healthcare professionals on sexual and reproductive health delivered using a blended learning approach (i.e., a combination of face‐to‐face learning and e‐learning approaches). Specifically, this systematic review will consider studies that assess the effectiveness of the following essential sexual and reproductive health interventions proposed by Starrs et al. (2018): (a) antenatal, childbirth and postnatal care, including emergency obstetric and newborn care, (b) safe abortion services and treatment of the complications of unsafe abortion, (c) prevention and treatment of malaria, tuberculosis, HIV and other sexually transmitted infections in pregnant women and (d) family planning. The manner of delivery, length and content of the educational programme may vary in each of the studies to be included as there is no standard sexual and reproductive health programme.

Interventions that are targeted at healthcare students will be excluded. Also, interventions targeted at healthcare practitioners working in high‐income countries will be excluded. Comparative conditions will include sexual and reproductive health blended learning compared with no intervention or with non‐blended learning approaches (such as conventional face‐to‐face learning, pure e‐learning and self‐directed learning). Included studies will be grouped according to the type of non‐blended learning approach that has been used as comparator/control and separate analyses will be done to assess the effectiveness of blended learning compared with different non‐blended learning approaches.

### 1.3 How the Intervention Might Work

A number of studies, including systematic reviews and meta‐analyses, have assessed whether blended learning interventions in healthcare education have an effect on the knowledge, skills and overall competence of healthcare practitioners. From these studies, it is evident that blended learning interventions, compared with nonblended learning interventions or no intervention, are effective in improving the competencies of learners (Al‐Shorbaji et al. 2015; Balakrishnan et al. 2021; Liu et al. 2016; McCutcheon et al. 2014; Rowe et al. 2012; Vallée et al. 2020).

However, there is limited evidence on studies that have examined the effectiveness of blended learning in training healthcare practitioners in sexual and reproductive health. Studies in this area have mostly focussed on evaluating the effectiveness of purely remote/online training for capacity strengthening in sexual and reproductive health. For example, a recent systematic review conducted by Perrotta et al. (2023) synthesised evidence on the effectiveness of remote education programmes to strengthen research capacity in sexual and reproductive health. The researchers included 6 studies, which involved a total of 2058 online learners, and found improvement in participants' research skills and knowledge, as well as improvement in attitudes and self‐efficacy towards research. However, the review only focussed on researchers as participants rather than healthcare professionals and assessed the impact of online learning rather than blended learning. Moreover, there is limited evidence on standardised proportions in which blended learning can effectively combine face‐to‐face learning with online instruction to produce a positive effect (Lazar et al. 2020; Owston and York 2018). However, according to Lazar et al. (2020) and Owston and York (2018), the proportion of the online learning component must be between 33% and 50%, or up to 80%.

Furthermore, while there is evidence to suggest strong similarities in research processes, terminology, practice and focus in blended learning education around the world (Spring and Graham 2017), the components of effective blended learning approaches are extremely heterogenous (Dziuban et al. 2018). For example, systematic reviews and meta‐analyses on blended learning have mostly included several approaches, such as simulations, online instructions, virtual and face‐to‐face interactions, emails, computer laboratories, scaffolding and mapping tools, interactive presentations and the use of online platforms such as Moodle and Google Classrooms. (Means et al. 2013).

In a systematic review to summarise the evidence on the different blended learning models that have been applied in introductory courses, Alammary (2019) identified five different components of blended learning, which include (1) face‐to‐face instructor‐led, where students attend a class and an instructor presents teaching and learning materials, with little engagement from students; (2) face‐to‐face collaboration, where students work together in class, for example, in discussion groups; (3) online instructor‐led, where instruction is delivered online and facilitated by an instructor who sets the pace (e.g., virtual classrooms); (4) online collaboration, where students work together online with their peers, for example, online learning communities and (5) online self‐paced, where students study at their own pace and time, and from their chosen location, for example, watching videos, online reading. This type of learning is sometimes called self‐directed learning.

Specific to LMICs, Byrne et al. (2016) evaluated the effectiveness of a blended learning approach in building the capacity of postgraduate Master's degree students in health research in Malawi. The findings revealed the need for effective collaboration and interaction among students and also between students and facilitators during online learning. Also, due to the relatively limited resources in LMICs, the research revealed the need to select appropriate technical tools and platforms that support online learning. Other factors that contributed to improving the effectiveness of a blended learning course included developing the online content of the course around low bandwidth availability, training facilitators and students on the tools used to deliver the online component, and involving a learning technologist to help navigate through challenges faced (Byrne et al. 2016).

**Figure 1:**
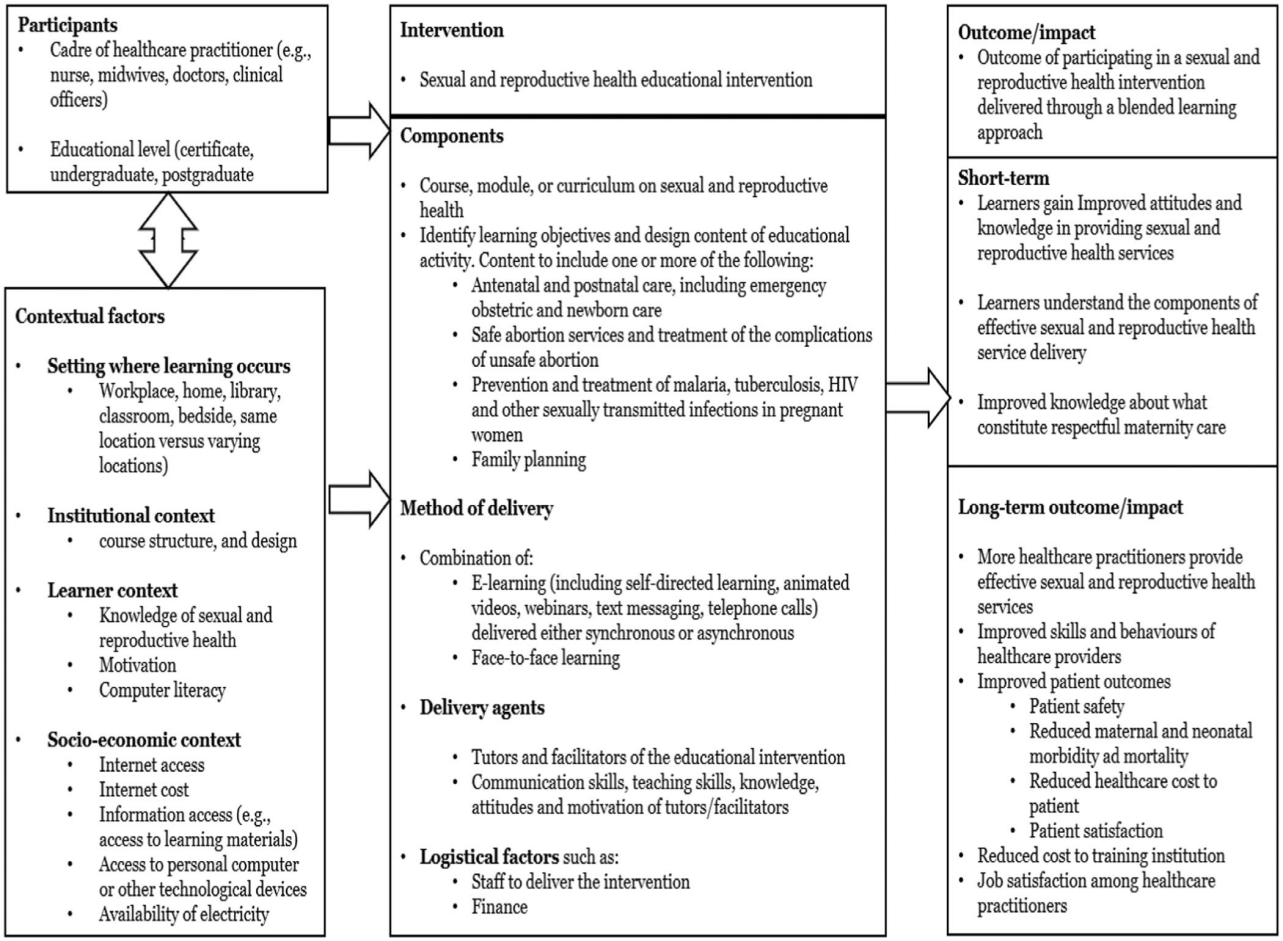
Systems‐based logic model of sexual and reproductive health education using blended learning.

Based on the above narrative, and our expertise in curriculum development and conducting capacity strengthening activities in LMICs, we have developed a systems‐based logic model (Figure 1) to indicate how using a blended learning approach in training healthcare practitioners in sexual and reproductive health in LMICs might lead to improved outcomes (including short and long‐term outcomes). The figure depicts the different elements, interactions and contextual factors that are essential to achieving desired outcomes.

### 1.4 Why It Is Important to Do This Review

To achieve the sustainable development goal agenda number three, targets 3.1, 3.2, 3.7 and 3.8, timely management and treatment of pregnancy and delivery‐related complications are essential. All antenatal, delivery and postnatal care must be attended by skilled healthcare practitioners (Say et al. 2014). Yet, LMICs experience significant crises in healthcare, and concerns regarding the readiness and quality of the healthcare workforce have been raised (Nicol et al. 2019). Hence, there is an urgent need for training for healthcare practitioners to equip them with the knowledge and skills needed to deliver quality healthcare services.

While several systematic reviews and meta‐analyses have assessed the effectiveness of blended learning, most of the studies were focused on high‐income countries and/or healthcare students. Moreover, systematic reviews in this area are limited to only some aspects of health education (e.g., pharmacy education) with no specific focus on the type of blended learning intervention. In Al‐Shorbaji et al.'s (2015) review, only 5 of the 49 studies involved were conducted in LMICs, making their findings not generalisable to low‐resource settings. This is because students' educational background, experiences and contexts may vary, which may have an impact on the overall findings of the study.

A more recent systematic review and meta‐analysis conducted by Balakrishnan et al. (2021) evaluated the effectiveness of blended learning approaches in improving the knowledge and skills of pharmacy students. Several other reviews have also evaluated the effectiveness of e‐learning in healthcare education (Cook et al. 2008; Jwayyed et al. 2011; Lahti and Välimäki 2009; Nicoll et al. 2018; Swaminathan et al. 2018). However, none of these reviews differentiated pure e‐learning from blended learning.

There is only one systematic review and meta‐analysis that evaluated the effectiveness of blended learning among healthcare professional learners compared with no intervention and with nonblended learning interventions (Liu et al. 2016). The target participants for Liu et al.'s (2016) systematic review were students, postgraduate trainees or practitioners in a profession directly related to animal or human health. The current systematic review is significantly different from Liu et al.'s (2016) review on three fronts. First, the present systematic review is unique as it focuses on sexual and reproductive health blended learning approaches and targets only healthcare practitioners (including nurses, midwives, medical doctors and nurse assistants) working in maternal and neonatal health services. Second, the current systematic review specifically assesses sexual and reproductive health blended learning approaches aimed at improving the competencies of healthcare practitioners, whereas Liu et al.'s (2016) systematic review had no specific focus on the type of blended learning approach. Third, the present systematic review only assesses sexual and reproductive health blended learning approaches that have been used in low‐ and middle‐income countries, whereas Liu et al.'s (2016) review had a global focus.

Findings from this systematic review will bridge the gaps in the current literature, as well as guide policy and practice, in designing effective interventions to improve care and future enquiry in this area of research and practice.

## 2 Objectives

The primary objective of this systematic review is to evaluate and synthesise both published and unpublished literature on the effectiveness of sexual and reproductive health blended learning approaches for capacity strengthening of healthcare practitioners in LMICs. Within this context, sexual and reproductive health interventions refer to any of the following four key interventions or services aimed at improving maternal and newborn health (Starrs et al. 2018): (a) antenatal, childbirth and postnatal care, including emergency obstetric and newborn care, (b) safe abortion services and treatment of the complications of unsafe abortion, (c) prevention and treatment of malaria, tuberculosis, HIV and other sexually transmitted infections in pregnant women and (d) family planning. In this systematic review, blended learning is defined as any teaching and learning method that combines face‐to‐face learning with e‐learning or online learning. The component of face‐to‐face and online learning may include any of the components identified by Alammary (2019): (1) face‐to‐face instructor‐led, where students attend a class and an instructor presents teaching and learning materials, with little engagement from students; (2) face‐to‐face collaboration, where students work together in class, for example, in discussion groups; (3) online instructor‐led, where instruction is delivered online and facilitated by an instructor who sets the pace (e.g., virtual classrooms); (4) online collaboration, where students work together online with their peers, for example, online learning communities; and (5) online self‐paced, where students study at their own pace and time, and from their chosen location, for example, watching videos, online reading.

Specifically, this systematic review will answer the following research questions:

1. What sexual and reproductive health blended learning approaches have been used in LMICs?
2. Does participating in sexual and reproductive health blended learning interventions alone (i.e., compared with no intervention) improve the effective provision of care among healthcare workers in LMICs?
3. Does participating in sexual and reproductive health blended learning interventions compared with non‐blended learning approaches (such as conventional face‐to‐face learning or pure e‐learning) facilitate the effective provision of care among healthcare workers in LMICs (measured by, e.g., self‐reports of effective maternal and neonatal care)?
4. What is the cost‐effectiveness of sexual and reproductive health blended learning compared with non‐blended learning approaches (i.e., face‐to‐face learning or e‐learning)?
5. What factors affect the effectiveness of sexual and reproductive health blended learning interventions (e.g., characteristics of participants, type of intervention, course content, setting and mode of delivery)?
6. Do sexual and reproductive health blended learning interventions targeted at healthcare practitioners working in LMICs lead to improvement in patient outcomes (e.g., reduced maternal and neonatal mortality, patient satisfaction reports)?

## 3 Methodology

### 3.1 Criteria for Considering Studies for This Review

#### 3.1.1 Types of Studies

The type of study designs to be included in this review will be considered based on recommendations by the Cochrane Collaboration's Effective Practice and Organisation of Care (EPOC) group (available at: https://epoc.cochrane.org/sites/epoc.cochrane.org/files/public/uploads/EPOC%20Study%20Designs%20About.pdf).

We will consider for inclusion all blended learning approaches used for delivering sexual and reproductive health interventions in which effectiveness is reported with either a comparator or a time series before and after evaluation. This includes, but not limited to, the following study designs:

- Randomised controlled trials: These are experimental studies in which participants are assigned to different interventions using a random method.
- Cluster randomised controlled trials: Experimental studies where groups of individuals (referred to as clusters) are assigned to different interventions using methods that are random.
- Non‐randomised controlled trials: these are experimental studies where participants are assigned to different groups that are being compared without using a random method.
- Controlled before‐after studies: These are study designs where decisions regarding the allocation of participants to comparison groups (i.e., the intervention and control group) are made by individuals other than the study investigators. In these study types, the outcomes of interest are measured before and after the intervention is implemented.

Based on recommendations by EPOC, for cluster randomised trials, non‐randomised cluster trials and controlled before‐after studies, we will exclude studies with only one intervention or control site, to avoid confounding related to the study site.

- Interrupted time series (ITS) designs: These are study designs that are used to measure the effect of an intervention in cases where it is impractical to apply randomisation or include a control group. It involves collecting multiple data points before and after implementing an intervention and measuring the effect of the intervention against the pre‐intervention data. We will exclude studies without clearly defined time points indicating when the intervention was implemented, as well as studies having two or less data points before and after the intervention.
- Cross‐sectional design: This is a type of observational study design that analyses data from a population or a pre‐specified subset at one given point in time.

Additionally, we will include studies with information about the implementation cost, as well as the kinds of sexual and reproductive health blended learning approaches that have been used in LMICs. Also, studies that measure healthcare practitioners' acceptability and satisfaction with sexual and reproductive health blended learning approaches will be considered for inclusion. These studies may be quantitative (e.g., retrospective and/or prospective cohort studies) or qualitative (e.g., focus groups, interviews, descriptive cross‐sectional studies). Findings from these studies will not be included in meta‐analysis, rather, they will be reported separately in a narrative or tabular form.

#### 3.1.2 Types of Participants

Participants in this systematic review will include healthcare practitioners working in low‐ and middle‐income countries. This will include, but not limited to, nurses, midwives, medical doctors, clinical officers and nurse assistants. Studies that include healthcare students will be excluded.

#### 3.1.3 Types of Interventions

This systematic review will consider primary studies that assess sexual and reproductive health blended learning approaches targeted at healthcare practitioners working in low‐ and middle‐income countries. In this systematic review, sexual and reproductive health blended learning approaches/interventions refer to educational programmes in sexual and reproductive health that are aimed at strengthening the capacity of healthcare practitioners through a combination of traditional face‐to‐face learning and e‐learning (including, but not limited to self‐directed learning, webinars, text messaging and telephone calls). The e‐learning training component may be delivered either synchronously or asynchronously.

Specifically, this systematic review will consider studies that assess the effectiveness of the following essential sexual and reproductive health interventions proposed by Starrs et al. (2018): (a) antenatal, childbirth and postnatal care, including emergency obstetric and newborn care, (b) safe abortion services and treatment of the complications of unsafe abortion, (c) prevention and treatment of malaria, tuberculosis, HIV and other sexually transmitted infections in pregnant women and (d) family planning. The manner of delivery, length and content of the educational programme may vary in each of the studies to be included as there is no standard sexual and reproductive health programme.

In this systematic review, sexual and reproductive health blended learning approaches that are targeted at healthcare students will be excluded. Also, sexual and reproductive health interventions targeted at healthcare practitioners working in high‐income countries will be excluded. Comparative conditions will include sexual and reproductive health blended learning compared with no intervention or with non‐blended learning approaches (such as conventional face‐to‐face learning and e‐learning).

#### 3.1.4 Types of Outcome Measures

To effectively evaluate the outcomes of blended learning in this review, we will use the Kirkpatrick model (Kirkpatrick 1959) to guide the process. The Kirkpatrick model is globally recognised for evaluating the outcomes of training and learning programmes. The model can be used for evaluating both formal and informal training and uses a four‐level rating criteria: (a) reaction, which measures the extent to which learners found the training favourable, engaging and relevant to their job role, and assesses outcomes such as acceptability, usefulness and satisfaction of the learners regarding the training programme; (b) learning, which assesses the extent to which the training led to improvement in participants' knowledge, skills, attitude, commitment and confidence; (c) behaviour, which measures whether training has truly led to improvement in outcomes and if participants are applying what they learned and (d) results, which measures the overall impact of the training based on the programme objectives, and assesses outcomes such as cost‐effectiveness, client satisfaction and improved quality of care.

In this review, we will apply all four levels of the Kirkpatrick model and consider studies that evaluate the following primary and secondary outcomes for inclusion.

##### 3.1.4.1 Primary Outcomes

To be considered for inclusion, studies must evaluate at least one of the following outcomes:

1. Level 1: Reaction

a. Healthcare practitioners' satisfaction with blended learning versus non‐blended learning approaches. This outcome may be measured by learner self‐reports, post‐course Likert scale questions, open‐ended questions and learner show of interest in the training programme (measured by looking at the percentage of learners that complete the training).

2. Level 2: Learning

a. Healthcare practitioners' knowledge: Measured by assessing knowledge scores using pre‐ and post‐course multiple‐choice questions, or any formal method of knowledge assessment presented in the included papers.
b. Healthcare practitioners' skills: Measured by evaluating learners' skills scores using pre‐ and post‐course practical tests (e.g., objective structured clinical examinations).
c. Healthcare practitioners' attitudes regarding effective maternal and neonatal care, measured by learner self‐reports, pre‐ and post‐course Likert‐scale questions.

Measurement of the above outcomes may be done using standardised or unstandardised tools. Examples of these include learner self‐reports, to evaluate learners' knowledge of the course, and practical tests to assess learners' skills and competence.

Level 3: Behaviour

a. Healthcare practitioners' behaviour regarding effective maternal and neonatal care: Measured through observation, reduction in maternal and neonatal deaths, self‐reports, patients' reported satisfaction of care and pre‐ and post‐test surveys.

##### 3.1.4.2 Secondary Outcomes

Level 4: Results

1. Factors that affect the effectiveness of sexual and reproductive health blended learning interventions, measured using open‐ended questions, post‐course Likert scale questionnaires and interviews.
2. The comparative cost of sexual and reproductive health blended learning and non‐blended learning interventions used in training healthcare practitioners in LMICs (this will include the sum of all the monetary costs involved in the training as well as the monetary cost of training each learner).
3. Patient outcomes measured using patient and healthcare practitioner satisfaction reports, reduced length of hospital stays, maternal and neonatal mortality rates and morbidity rates.

#### 3.1.5 Duration of Follow‐Up

In this systematic review, no limit will be placed on the duration of follow‐up. This is to allow studies with either long‐ or short‐term duration to be eligible for inclusion.

#### 3.1.6 Types of Settings

Primary studies from all geographical locations of LMICs will be considered for inclusion in this systematic review. However, due to language translation issues, only eligible studies whose full texts are in the English language will be included in the data synthesis.

#### 3.1.7 Time

There will be no limit to the publication date of included studies.

### 3.2 Search Methods for Identification of Studies

We will develop a comprehensive search strategy with the aim to identify eligible published and unpublished papers for inclusion in this systematic review. The search strategy will consist of a combination of keywords and key terms related to the population, intervention, outcomes and study designs. The following sources will be searched for eligible studies.

#### 3.2.1 Electronic Database Search

The following databases will be searched:

- Medline.
- CINAHL.
- Global Health.
- ERIC.
- PubMed.
- Cochrane.
- Web of Science.
- Scopus.
- University of Liverpool's Discover database.
- Proquest.
- ScienceDirect.

Supporting Information S1: Appendix 1 presents the search strategy for the Medline database searched via the EBSCO platform. We will modify the search strategy and terms for the other databases.

#### 3.2.2 Searching Other Resources

1. Web search using the following search engines:
  - Google
  - Google Scholar
2. Grey literature search:
  - OpenGrey (System for information on grey literature)
  - The Society for Research on Educational Effectiveness
3. Screen the reference list of previously conducted systematic reviews, meta‐analyses and eligible primary studies to identify relevant studies.
4. Contact leading authors.

The corresponding authors of identified eligible abstracts, whose full texts are unavailable will be contacted to request for full text reports.

### 3.3 Management of References

The Endnote (20) software will be used for reference management. Search results will be imported directly into the Endnote library. Where this is not possible, search outputs will be entered manually into the Endnote library.

### 3.4 Criteria for Determination of Independent Findings

Non‐independence of results may occur if multiple measures of the same outcome are reported in a single study. Non‐independence can also occur if more than one article reports on study findings that were all based on the same sample. Where a similar outcome is measured at multiple time points (e.g., 12 months follow‐up, 6 months follow‐up, 3 months follow‐up, immediate post‐test), data analysis will focus on the time point closest to the end of the intervention period. Where there are multiple reports of the same outcome, we will calculate an average weighted effect size within each study for each outcome. Where multiple reports of the same study are identified, data will only be extracted once from the most complete and detailed report. Multiple publications will be identified by scrutinising characteristics, such as author names, sample size, study time frame and intervention programmes.

### 3.5 Data Collection and Analysis

#### 3.5.1 Selection of Studies

Two review authors (E.A.K. and A.L. or F.M. and Z.S./Y.S.) will independently screen search outputs for eligible studies using Covidence (a web‐based platform). We will first screen the title and abstract of search results, followed by the full texts of seemingly eligible studies. These articles will be screened using the selection criteria indicated above. Disagreements between reviewers will be resolved through discussion and by consulting a third author (C.A., S.A.W. or N.F.).

#### 3.5.2 Data Extraction and Management

Data extraction will be conducted by two reviewers (E.A.K. and Z.S. or F.M./A.L. and Y.S.) in Covidence, using a data extraction tool adapted from the Cochrane data collection form for intervention reviews for RCTs and non‐RCTs (see Supporting Information S2: Appendix 2). The data extraction tool will first be pilot‐tested on 10% of the included articles and amended where necessary. Data and information to be extracted from included studies will include characteristics of the population, details of the intervention and the comparison group(s), group sizes, study design, outcome measures relevant to the review questions and objectives and their timing; estimates with confidence intervals (or other measure of variability) and numbers included in each statistic, for each group and each comparison, with confidence intervals. Disagreements between reviewers will be resolved through discussions or by consulting another review author (C.A., S.A.W. or N.F.).

#### 3.5.3 Assessment of Risk of Bias in Included Studies

Risk of bias of included randomised studies will be assessed using the Cochrane Collaboration's risk of bias tool (Higgins et al. 2019). The tool comprises five domains covering all forms of bias that can impact findings from randomised trials, with each domain having a set of signalling questions. Each signalling question is judged as having either ‘high’, ‘some concerns’ or ‘low’ risk of bias. For individually randomised trials, the five risk of bias domains are as follows:

1. Bias arising from the randomisation process,
2. Bias due to deviations from intended interventions,
3. Bias due to missing outcome data,
4. Bias in the measurement of the outcome,
5. Bias in the selection of the reported result.

For cluster randomised trials, Higgins et al. (2019) suggest an additional domain, which is bias arising from the identification or recruitment of individual participants within clusters. Thus, for included cluster randomised trials, in addition to the above‐listed risk of bias domains for individually randomised trials, we will assess the risk of bias using the signalling questions relating to bias arising from the identification or recruitment of individual participants within clusters. Risk of bias assessment will be conducted independently by two review authors (either E.A.K. and Z.S. or F.M./A.L. and Y.S.) and disagreements will be resolved either through discussion among reviewers or by contacting a third review author (C.A., S.A.W. or N.F.).

For non‐randomised study designs, we will assess the risk of bias using the Risk of Bias in Non‐randomised Studies – of Interventions (ROBINS‐I) tool. Non‐randomised study designs to be considered for inclusion in this review include observational studies such as case‐control studies and cohort studies where intervention groups are assigned during the course of usual treatment decisions as well as quasi‐randomised studies in which the method used in assigning participants falls short of complete randomisation (Sterne et al. 2016). The ROBINS‐I tool will also be used to assess the risk of bias in study designs, such as cross‐sectional studies, ITS and controlled before‐after studies.

We will use the signalling questions in the following seven domains included in the ROBINS‐I tool to assess the risk of bias of included non‐randomised studies:

*Pre‐intervention*

1. Bias due to confounding.
2. Bias in the selection of participants for the study.

*At intervention*

3. Bias in the classification of intervention.

*Post‐intervention*

4. Bias due to deviations from intended interventions.
5. Bias due to missing data.
6. Bias in the measurement of outcomes.
7. Bias in the selection of the reported result.

The signalling questions of the above domains will be judged using five categories: (a) low risk of bias, (b) moderate risk of bias, (c) serious risk of bias, (d) critical risk of bias and (e) no information.

For qualitative studies, we will use the Critical Appraisal Skills Programme (CASP) for qualitative studies to assess study quality. The CASP checklist is a standardised tool for assessing the methodological quality of qualitative studies. It contains 10 questions, which assess the validity, reliability and transferability of the study results.

#### 3.5.4 Measures of Treatment Effect

##### 3.5.4.1 Continuous Data

We will estimate the mean difference with a 95% confidence interval for each comparison involving scores, where the same type of assessment tool has been used in measuring outcomes. Examples include maternal and neonatal health knowledge, attitudes and skills, as well as statisfaction with and cost of training. If outcomes are measured using different types of tools, we will estimate standardised mean differences (SMDs) as the effect size metric based on Hedges' *g*, calculated as follows:

SMD = Difference in mean outcome between groups/Standard deviation of outcome among participants.

##### 3.5.4.2 Dichotomous Data

For dichotomous data, we will calculate risk ratios with 95% confidence intervals. Dichotomous outcomes in this review include patient outcomes, and factors affecting the effectiveness of sexual and reproductive health blended learning interventions. For the purposes of meta‐analysis, risk ratios will be converted to SMD, using David Wilson's practical effect size calculator. We will report outcomes that have not been measured numerically in a qualitative manner.

##### 3.5.4.3 Studies With Multiple Groups

For studies with two or more intervention groups compared with one control group and all the interventions are regarded as relevant to the current review, we will use the following options: (a) if the intervention groups are dissimilar, we will divide the sample size of the control group into two (or more depending on the number of intervention groups) and compare this with the intervention groups, (b) where the intervention groups are similar, we will treat the two groups as a single group. Thus, we will conduct two effect size estimates in this review. This will ensure that participants in control groups are not ‘double counted’ (Higgins and Green 2011).

##### 3.5.4.4 Studies Reporting Multiple Outcome Measures

Where multiple outcome measures are reported for the same participants, leading to non‐independent effect sizes, we will use the Robust Variance Estimation approach (Hedges et al. 2010) to address this. The Robust Variance Estimation can be used to deal with non‐independent effect sizes. It incorporates small sample size corrections, which can reduce inflated type 1 errors as a result of clustering in cluster randomised studies.

#### 3.5.5 Unit of Analysis Issues

In this review, it is expected that included studies may involve either groups (clusters) of participants or individual participants as units of analysis. For eligible cluster randomised trials that have used appropriate analytic methods to properly account for the cluster design, we will include the effect estimates and their standard errors in meta‐analysis. However, we will employ standard conversion criteria recommended by Higgins and Green (2011), if cluster randomised trials included in this review have not applied appropriate methods (e.g., using multi‐level modelling or robust standard errors) to control for clustering effect.

#### 3.5.6 Dealing With Missing Data

We will contact the first author of eligible studies with incomplete reports on data to request relevant information that is missing from the report. If requested data is not provided, we will determine whether data is ‘missing at random’ (i.e., if the fact that they are missing is unrelated to the actual values of the missing data) or ‘missing not at random’ (i.e., if the fact that they are missing is related to the actual missing data). We will then apply the following two options: (1) if data is missing at random, we will conduct data analysis based on the available data; (2) if data is missing not at random, we will impute the missing data with replacement values, which will be treated as if they were observed (e.g., imputing the mean based on predicted values from a regression analysis, or imputing an assumed outcome as good or poor).

#### 3.5.7 Assessment of Reporting Biases

Studies included in this review will be assessed for reporting bias to determine any discrepancies between reported outcomes and measured outcomes. We will consider studies as having a low risk of bias if pre‐specified outcomes have been clearly reported in the results section.

#### 3.5.8 Data Synthesis

Included studies will be synthesised using narrative and statistical methods. For each outcome and comparison, a random‐effects meta‐analysis will be performed if there are two or more eligible studies that can be appropriately grouped together. Analysis will be performed using the meta summary command in Stata version 17.0. We will analyse data by type of sexual and reproductive health on which outcome is reported (e.g., antenatal, childbirth and postnatal care), and conduct separate meta‐analyses for primary and secondary outcomes.

The decision to combine studies in meta‐analysis will be based on whether there are multiple eligible studies that share similar characteristics. These characteristics may include the type of intervention (e.g., antenatal care) and the expected outcome(s) of the intervention (e.g., improved knowledge). Means and standard deviations reported in the included studies will be converted to SMDs based on the Hedges' *g* effect size. This is because we anticipate that most eligible studies assessing the same outcomes (e.g., knowledge, skills and behaviour) are likely to use different tools in measuring them. Effect estimates will be reported with 95% confidence intervals.

We will conduct thematic content analysis using the method recommended by Braun and Clarke (2006), to synthesise qualitative data. This will allow us to identify patterns within qualitative data. This will involve three main stages:

a. Line by line inductive coding,
b. Development of descriptive themes,
c. Development of analytical themes.

#### 3.5.9 Subgroup Analysis and Investigation of Heterogeneity

We will assess heterogeneity by comparing factors, such as characteristics of participants, type of intervention, type of outcome measures and control comparators, as well as factors affecting the effectiveness of interventions.

Educational heterogeneity will be assessed by presenting, in a tabular format, the population characteristics, intervention design, content and delivery, as well as outcome(s) assessed, and methods used in assessing outcomes. We will also assess and report the educational context where the intervention was delivered (see Figure 1).

We will explore methodological heterogeneity by clearly presenting the risk of bias results for each included study as well as the different study designs that have been employed by included studies.

Statistical heterogeneity will be assessed visually using forest and Galbraith plots and quantified using the *I* ^2^ statistic. The *I* ^2^ statistic defines the estimated proportion of variation that is due to heterogeneity rather than sampling error. By convention, *I* ^2^ values of 25% are considered low, 50% moderate and 75% high. We will supplement this assessment with Cochrane's homogeneity test, using the 5% level to determine the significance of heterogeneity. We will also present *τ* ^2^, together with its confidence interval, to indicate the magnitude of heterogeneity between studies. We will conduct sensitivity and subgroup analysis to determine the possible sources of heterogeneity. Where there is high heterogeneity between included studies, data will not be pooled, but rather we will present results of individual studies in a narrative and tabular manner. This will include a clear description of the population, intervention, comparisons, outcome measured, outcome assessment method and results.

#### 3.5.10 Sensitivity Analysis

Sensitivity analysis will be done to establish whether the overall results of data synthesis will be influenced by removing any of the following:

- Unpublished studies,
- Studies with missing data,
- Studies with high risks of bias.

## Author Contributions

- Content, systematic review methods and information retrieval: Dr. Elizabeth A. Kumah is a registered general nurse who has worked mainly in the critical care setting as a nurse supervisor and patient advocate. Elizabeth has extensive experience working in low‐resource settings as a healthcare practitioner. She has been actively engaged in teaching healthcare students in the clinical setting and serving as a mentor. She brings methodological, information retrieval and content expertise relating to maternal and neonatal health in low‐ and‐middle‐income settings, as well as the development of educational programmes targeted at healthcare students and professionals. Elizabeth's area of expertise is in evidence‐informed/evidence‐based practice, and patient safety and quality, which she has applied effectively within both research and educational programmes. Elizabeth has led a Campbell systematic review on evidence‐informed practice and evidence‐based practice educational interventions, which has recently been published by the Campbell Collaboration. She has also been involved in several other systematic and scoping reviews, both as a lead author and co‐author. She is passionate about improving the standard of patient care and patient outcomes, which she believes could be achieved through capacity strengthening of healthcare practitioners, among others.
- Content, systematic review methods and information retrieval: Dr. Florence Mgawadere is a nurse‐midwife and a public health specialist with over 17 years of experience in teaching, conducting research, designing and leading on implementation and evaluation of SRH programmes in low‐ and middle‐income countries (LMICs). Florence brings to the team specialist skills in qualitative and quantitative methods applied to health services research, research synthesis and research uptake relevant to health problems in LMICs. She has conducted and published more than three systematic reviews, including planning, assembling a team, writing a protocol, searching the evidence, information retrieval, analysing and synthesising results.
- Content and systematic review methods: Dr. Alice Ladur is a public health specialist with extensive experience in teaching, research and implementation of maternal health programmes in low‐and‐middle‐income countries (LMICs). She brings to the team expertise in narrative synthesis and qualitative research methods. Alice has published narrative reviews, scoping reviews and qualitative research in Sexual and Reproductive Health.
- Content: Zainab Suleiman is a lecturer in nursing at the State University of Zanzibar, Tanzania. She is currently a PhD student at the Liverpool School of Tropical Medicine, researching ways to improve in‐service reproductive health capacity strengthening programmes by comparing the effectiveness of face‐to‐face learning and blended learning approaches in low‐ and middle‐income countries, focussing specifically on Nigeria, Kenya, and Tanzania. Zainab brings to the team expertise in sexual and reproductive health service delivery in low‐ and middle‐income countries.
- Content: Yusupha Sanyang is a registered nurse in the Gambia, and is currently a PhD student at the Liverpool School of Tropical Medicine. Yusupha brings to the team expertise in sexual and reproductive health service delivery in low‐ and middle‐income countries.
- Statistical Analysis: Dr. Sarah A. White is a statistician with over twenty years of experience working in biomedical statistics while residing in Africa, using her knowledge and skills to train others at both undergraduate and postgraduate levels and to support research. She currently works in the Emergency Obstetric and Quality of Care unit at the Liverpool School of Tropical Medicine, supporting research in maternal and newborn health. She has experience in designing and analysing randomised controlled clinical trials, including trials measuring longitudinal or survival‐type outcomes, cluster randomised trials, cross‐sectional surveys and method comparison studies. Sarah brings statistical expertise to the team.
- Content: Dr. Nicholas Furtado is a medical advisor at the Global Fund and has expertise and experience in reproductive, maternal, newborn and child health as well as health systems strengthening in low‐ and middle‐income countries. Nicholas brings to the team content expertise in sexual and reproductive health intervention design and delivery in low‐ and middle‐income countries.
- Content and systematic review methods: Professor Charles Ameh leads the project, ‘quality improvement of integrated HIV, tuberculosis and malaria services in antenatal and postnatal care’, funded by the Takaeda Pharmaceutical Company through the Global Fund. Charles has extensive expertise and experience in emergency obstetric care and quality of care (including maternal and newborn care, sexual and reproductive health and quality improvement of healthcare services). He has conducted and published several systematic and scoping reviews, both as a lead author and co‐author. Charles brings to the team knowledge and skills in systematic review methods, as well as expertise in sexual and reproductive health intervention design and delivery in low‐ and middle‐income countries.

## Conflicts of Interest

The authors declare no conflicts of interest.

## Sources of Support

### External Sources

Takaeda Pharmaceutical Company through the Global Fund, Japan.

This systematic review forms part of the work supported by the Takaeda Pharmaceutical Company through the Global Fund.

## Author Declaration

### Authors' Responsibilities

By completing this form, you accept responsibility for preparing, maintaining and updating the review in accordance with Campbell Collaboration policy. Campbell will provide as much support as possible to assist with the preparation of the review.

A draft review must be submitted to the relevant Coordinating Group within 2 years of protocol publication. If drafts are not submitted before the agreed deadlines, or if we are unable to contact you for an extended period, the relevant Coordinating Group has the right to de‐register the title or transfer the title to alternative authors. The Coordinating Group also has the right to de‐register or transfer the title if it does not meet the standards of the Coordinating Group and/or Campbell.

You accept responsibility for maintaining the review in light of new evidence, comments and criticisms, and other developments, and updating the review at least once every 5 years, or, if requested, transferring responsibility for maintaining the review to others as agreed with the Coordinating Group.

### Publication in the Campbell Library

The support of the Coordinating Group in preparing your review is conditional upon your agreement to publish the protocol, finished review, and subsequent updates in the Campbell Library. Campbell places no restrictions on publication of the findings of a Campbell systematic review in a more abbreviated form as a journal article either before or after the publication of the monograph version in Campbell Systematic Reviews. Some journals, however, have restrictions that preclude publication of findings that have been, or will be, reported elsewhere and authors considering publication in such a journal should be aware of possible conflict with publication of the monograph version in Campbell Systematic Reviews. Publication in a journal after publication or in press status in Campbell Systematic Reviews should acknowledge the Campbell version and include a citation to it. Note that systematic reviews published in Campbell Systematic Reviews and co‐registered with Cochrane may have additional requirements or restrictions for co‐publication. Review authors accept responsibility for meeting any co‐publication requirements.

*I understand the commitment required to undertake a Campbell review and agree to publish in the Campbell Library. Signed on behalf of the authors:*

**Table:** 

Form completed by: Elizabeth Adjoa Kumah	Date: 30th May 2024

## Supporting information

Supporting information.

Supporting information.
