# Supplementary material for: PROTOCOL: Effectiveness of Sexual and Reproductive Health Blended Learning Approaches for Capacity Strengthening of Health Professionals in Low‐ and Middle‐Income Countries: A Systematic Review
Source: Campbell Syst Rev. 2025 Mar 11;21(1):e70028. doi: 10.1002/cl2.70028 (PMC11894264; doi:10.1002/cl2.70028)
Supplement: Supplementary file 1 — Supporting information. [file CL2-21-e70028-s001.docx]

Appendix 1

|  | **Sexual and Reproductive Health** |  |
| --- | --- | --- |
| #1 | “sexual and reproductive health” OR “reproductive medicine” OR “maternal welfare” OR “maternal wellbeing” OR “maternal well-being” OR “infant welfare” OR “infant wellbeing” OR “infant well-being” OR “child welfare” OR “child wellbeing” OR “child well-being” OR pregnan* OR childbirth OR obstetric* OR parturition OR antenatal OR prenatal OR postnatal OR postpartum OR puerperium OR perinatal OR “family planning” OR “planned pregnanc*” OR “sex* education” OR “birth control” OR “fertility control” OR contracepti* OR condom* OR abort* OR post-abort* OR postabort* OR STD OR STI | Title/ Abstract |
| #2 | Reproductive Health OR Sexual Health OR Reproductive Health Services OR Reproductive Medicine OR Maternal Health OR Maternal Health Services OR Maternal-Child Health Services OR Maternal Welfare OR Child Health OR Child Health Services OR Infant Health OR Infant Welfare OR Womens Health OR Women Health Services OR Pregnant Women OR Pregnancy OR Pregnancy Complications OR Obstetrics OR Delivery, Obstetric OR Labor, Obstetric OR Obstetric Labour Complications OR Obstetric Nursing OR Obstetric Labor, Premature OR Premature Birth OR Infant, Premature OR Infant, Newborn OR Prenatal Care OR Postnatal Care OR Parturition OR Postpartum Period OR Sexually Transmitted Diseases OR Family Planning Services OR Sex Education OR Contraception OR Contraceptive Agents OR Condoms OR Abortion, Induced OR Abortion, Criminal OR Abortion, Legal OR Abortion, Therapeutic | MeSH Terms |
| #3 | ((sexual OR reproductive OR maternal OR child* OR neonat* OR infant* OR newborn OR mother* OR women* OR woman*) N1 health*) | Title/ Abstract |
| #4 | (terminat* N2 pregnanc*) | Title/ Abstract |
| #5 | (“sexually transmitted”) N2 (infection* OR disease*) | Title/ Abstract |
| #6 | hiv OR hiv-1* OR hiv-2* OR hiv1 OR hiv2 OR “HIV infect*” OR “human immunodeficiency virus” OR “human immunedeficiency virus” OR “human immuno-deficiency virus” OR “human immune-deficiency virus” OR “acquired immunodeficiency syndrome” OR “acquired immunedeficiency syndrome” OR “acquired immuno-deficiency syndrome” OR “acquired immune-deficiency syndrome” OR tuberculos* OR tb OR Koch* OR XDR-TB OR MDR-TB OR “XDR TB” OR “MDR TB” OR “pulmonary consumption” OR “pulmonary phthisis” OR malaria OR “plasmodium infection*” | Title/ Abstract |
| #7 | HIV OR HIV Infections OR Tuberculosis OR Tuberculosis, Pulmonary OR Mycobacterium Tuberculosis OR Tuberculosis, Multidrug- Resistant OR Extensively Drug-Resistant Tuberculosis OR Latent Tuberculosis OR Tuberculosis, Pleural OR Malaria | MeSH Terms |
| #8 | pregnan* | Title/ Abstract |
| #9 | Pregnancy OR Pregnant Women | MeSH Terms |
| #10 | #1 OR #2 OR #3 OR #4 OR #5 |  |
| #11 | #6 OR #7 |  |
| #12 | #8 OR #9 |  |
| #13 | #11 AND #12 |  |
| **#14** | **#10 OR #13** | **2,318,168** |
|  |  |  |
|  | **Blended Learning** |  |
| #1 | (blended OR hybrid OR online OR on-line OR electronic OR computer-aided OR “computer aided” OR computer-assisted OR “computer assisted” OR “computer-based” OR “computer based” OR flipped OR digital OR technology-enhanced OR “technology enhanced” OR technology-assisted OR “technology assisted” OR distance OR web* OR mobile OR computerised OR computerized OR virtual OR internet* OR video* OR “instant messag*” OR iphone OR iPad OR remote OR ) N4 (learning OR educat* OR program* OR training OR course* OR teach* OR instruct* OR curricul* OR module OR approach OR tutorial OR tuition OR workshop OR work-shop OR class* OR in-service OR inservice OR seminar) | Title/ Abstract |
| #2 | e-learning OR elearning OR “e learning” OR “e train*” OR e-instruction OR e-education OR “e curricul*” OR “e program*” OR m-learning OR mlearning | Title/ Abstract |
| #3 | Education, Distance OR Computer Assisted Instruction | MeSH Terms |
| #4 | Internet OR Cell Phone OR Mobile Applications OR Computers, Handheld | MeSH Terms |
| #5 | Education | MeSH Terms |
| #6 | #1 OR #2 OR #3 |  |
| #7 | #4 AND #5 |  |
| **#8** | **#6 OR #7** | **141,983** |
|  |  |  |
|  | **Healthcare Practitioners** |  |
| #1 | midwife OR midwives OR nurs* OR doctor* OR medic* OR physician* OR clinician* OR surgeon* OR “birth attendant*” | Title/ Abstract |
| #2 | Nurse Midwives OR Nursing Assistants OR Nursing OR Midwifery OR Health Personnel OR Physicians OR Physicians, Women OR Community Health Workers OR Medical Staff OR Medical Staff, Hospital OR Health Occupations | MeSH Terms |
| #3 | (health* N1 (assistant* OR profession* OR practitioner* OR worker* OR provider* OR personnel OR aide*)) | Title/ Abstract |
| #4 | #1 OR #2 OR #3 |  |
| **#5** | **#1 OR #2 OR #3** | **4,909,642** |
|  |  |  |
|  | **Low to Middle Income Countries*** |  |
| #1 | Developing Countries | MeSH Terms |
| #2 | Africa or Asia or Caribbean or West Indies or South America or Latin America or Central America | No fields selected |
| #3 | Afghanistan or Albania or Algeria or Angola or Antigua or Barbuda or Argentina or Armenia or Armenian or Aruba or Azerbaijan or Bahrain or Bangladesh or Barbados or Benin or Byelarus or Byelorussian or Belarus or Belorussian or Belorussia or Belize or Bhutan or Bolivia or Bosnia or Herzegovina or Hercegovina or Botswana or Brasil or Brazil or Bulgaria or Burkina Faso or Burkina Fasso or Upper Volta or Burundi or Urundi or Cambodia or Khmer Republic or Kampuchea or Cameroon or Cameroons or Cameron or Camerons or Cape Verde or Central African Republic or Chad or Chile or China or Colombia or Comoros or Comoro Islands or Comores or Mayotte or Congo or Zaire or Costa Rica or Cote d'Ivoire or Ivory Coast or Croatia or Cuba or Cyprus or Czechoslovakia or Czech Republic or Slovakia or Slovak Republic or Djibouti or French Somaliland or Dominica or Dominican Republic or East Timor or East Timur or Timor Leste or Ecuador or Egypt or United Arab Republic or El Salvador or Eritrea or Estonia or Ethiopia or Fiji or Gabon or Gabonese Republic or Gambia or Gaza or Georgia Republic or Georgian Republic or Ghana or Gold Coast or Greece or Grenada or Guatemala or Guinea or Guam or Guiana or Guyana or Haiti or Honduras or Hungary or India or Maldives or Indonesia or Iran or Iraq or Isle of Man or Jamaica or Jordan or Kazakhstan or Kazakh or Kenya or Kiribati or Korea or Kosovo or Kyrgyzstan or Kirghizia or Kyrgyz Republic or Kirghiz or Kirgizstan or Lao PDR or Laos or Latvia or Lebanon or Lesotho or Basutoland or Liberia or Libya or Lithuania or Macedonia or Madagascar or Malagasy Republic or Malaysia or Malaya or Malay or Sabah or Sarawak or Malawi or Nyasaland or Mali or Malta or Marshall Islands or Mauritania or Mauritius or Agalega Islands or Mexico or Micronesia or Middle East or Moldova or Moldovia or Moldovian or Mongolia or Montenegro or Morocco or Ifni or Mozambique or Myanmar or Myanma or Burma or Namibia or Nepal or Netherlands Antilles or New Caledonia or Nicaragua or Niger or Nigeria or Northern Mariana Islands or Oman or Muscat or Pakistan or Palau or Palestine or Panama or Paraguay or Peru or Philippines or Philipines or Phillipines or Phillippines or Poland or Portugal or Puerto Rico or Romania or Rumania or Roumania or Russia or Russian or Rwanda or Ruanda or Saint Kitts or St Kitts or Nevis or Saint Lucia or St Lucia or Saint Vincent or St Vincent or Grenadines or Samoa or Samoan Islands or Navigator Island or Navigator Islands or Sao Tome or Saudi Arabia or Senegal or Serbia or Montenegro or Seychelles or Sierra Leone or Slovenia or Sri Lanka or Ceylon or Solomon Islands or Somalia or South Africa or Sudan or Suriname or Surinam or Swaziland or Syria or Tajikistan or Tadzhikistan or Tadjikistan or Tadzhik or Tanzania or Thailand or Togo or Togolese Republic or Tonga or Trinidad or Tobago or Tunisia or Turkey or Turkmenistan or Turkmen or Uganda or Ukraine or Uruguay or USSR or Soviet Union or Union of Soviet Socialist Republics or Uzbekistan or Uzbek or Vanuatu or New Hebrides or Venezuela or Vietnam or Viet Nam or West Bank or Yemen or Yugoslavia or Zambia or Zimbabwe or Rhodesia | No fields selected |
| #4 | ((developing or less* developed or under developed or underdeveloped or middle income or low* income or underserved or under served or deprived or poor*) N1 (countr* or nation? or population? or world)) | No fields selected |
| #5 | ((developing or less* developed or under developed or underdeveloped or middle income or low* income) N1 (economy or economies)) | No fields selected |
| #6 | (low* N1 (gdp or gnp or gross domestic or gross national)) | No fields selected |
| #7 | (low N3 middle N3 countr*) | No fields selected |
| #8 | (lmic or lmics or third world or lami countr*) | No fields selected |
| #9 | transitional countr* | No fields selected |
| #10 | ((high burden or high-burden or countdown) N1 countr*) | No fields selected |
| **#11** | **#1 OR #2 OR #3 OR #4 OR #5 OR #6 O #7 OR #8 OR 9 OR 10** | **7,734,272** |
|  | **#14 AND #8 AND #5 AND #11** | **1,147** |
