# Supplementary material for: PROTOCOL: Effectiveness of Sexual and Reproductive Health Blended Learning Approaches for Capacity Strengthening of Health Professionals in Low‐ and Middle‐Income Countries: A Systematic Review
Source: Campbell Syst Rev. 2025 Mar 11;21(1):e70028. doi: 10.1002/cl2.70028 (PMC11894264; doi:10.1002/cl2.70028)
Supplement: Supplementary file 2 — Supporting information. [file CL2-21-e70028-s002.doc]

**Data extraction form**

**Systematic review title**: Effectiveness of Sexual and Reproductive Health Blended Learning Approaches for Capacity Strengthening of Health Professionals in Low- And Middle-Income Countries: A Systematic Review

## This data extraction template has been developed by adopting and customising the Cochrane Collaboration’s ‘data collection form for intervention review for RCTs and non-RCTs. We have adapted the tool by removing irrelevant items from the original template and added some new items to the template.

## Notes on using data extraction form:

- Be consistent in the order and style you use to describe the information for each report.
- Record any missing information as unclear or not described, to make it clear that the information was not found in the study report(s), not that you forgot to extract it.
- Include any instructions and decision rules on the data collection form, or in an accompanying document. It is important to practice using the form and give training to any other authors using the form.

| Title of the study/article/paper/report from which data is being collected |  |
| --- | --- |
| Study ID *(surname of first author and year first full report of study was published e.g. Smith 2001)* |  |
| Report ID |  |
| Report ID of other reports of this study (e.g., follow-up studies, duplicate publications) |  |
| Notes | |

# General Information

| Date form completed *(dd/mm/yyyy)* |  |
| --- | --- |
| Name/ID of person extracting data |  |
| Reference citation |  |
| Study author contact details |  |
| Publication type *(e.g. full report, abstract, letter)* |  |
| Study funding source *(including role of funders)* |  |
| Possible conflicts of interest *(for study authors)* |  |
| Notes: | |

# Characteristics of included studies

## Methods

|  | **Descriptions as stated in report/paper** | | **Location in text or source** *(pg & ¶/fig/table/other)* |
| --- | --- | --- | --- |
| **Aim/objective/research question of study** |  | |  |
| **Study Design** *(e.g. RCT, non-RCT, cohort study, case-control study, cross-sectional study, interrupted time series with a control group or a comparative study with concurrent controls, qualitative study)* |  | |  |
| **Unit of allocation**  *(by individuals, cluster/ groups or body parts)* |  | |  |
| **Start date** |  | |  |
| **End date** |  | |  |
| **Duration of participation**  *(from recruitment to last follow-up)* |  | |  |
| **Ethical approval needed/ obtained for study** | Yes No Unclear |  |  |
| **Notes:** | | | |

## Participants

|  | Description  *Include comparative information for each intervention or comparison group if available* | | Location in text or source *(pg & ¶/fig/table/other)* |
| --- | --- | --- | --- |
| Population description  *(from which study participants are drawn)* |  | |  |
| Setting  *(including location and social context)* |  | |  |
| Inclusion criteria |  | |  |
| Exclusion criteria |  | |  |
| Method of recruitment of participants *(e.g. phone, mail, clinic patients)* |  | |  |
| Informed consent obtained | Yes No Unclear |  |  |
| Total no. randomised  *(or total pop. at start of study for NRCTs)* |  | |  |
| Clusters  *(if applicable, no., type, no. people per cluster)* |  | |  |
| Notes: | | | |

## Intervention groups

*Copy and paste table for each intervention and comparison group*

Intervention Group 1

|  | Description as stated in report/paper | Location in text or source *(pg & ¶/fig/table/other)* |
| --- | --- | --- |
| Group name |  |  |
| No. randomised to group  *(specify whether no. people or clusters)* |  |  |
| Theoretical basis *(include key references)* |  |  |
| Description *(include sufficient detail for replication, e.g. content, dose, components)* |  |  |
| Duration of treatment period |  |  |
| Timing *(e.g. frequency, duration of each episode)* |  |  |
| Delivery *(e.g. mechanism, medium, intensity, fidelity)* |  |  |
| Providers  *(e.g. no., profession, training, ethnicity etc. if relevant)* |  |  |
| Co-interventions |  |  |
| Economic information *(i.e. intervention cost, changes in other costs as result of intervention)* |  |  |
| Resource requirements  *(e.g. staff numbers, cold chain, equipment)* |  |  |
| Integrity of delivery |  |  |
| Compliance |  |  |
| Notes: | | |

## Outcomes

*Copy and paste table for each outcome.*

Outcome 1

|  | Description as stated in report/paper | | Location in text or source *(pg & ¶/fig/table/other)* |
| --- | --- | --- | --- |
| Outcome name |  | |  |
| Time points measured  *(specify whether from start or end of intervention)* |  | |  |
| Time points reported |  | |  |
| Outcome definition *(with diagnostic criteria if relevant)* |  | |  |
| Person measuring/ reporting |  | |  |
| Unit of measurement  *(if relevant)* |  | |  |
| Scales: upper and lower limits *(indicate whether high or low score is good)* |  | |  |
| Is outcome/tool validated? | Yes No Unclear |  |  |
| Imputation of missing data *(e.g. assumptions made for ITT analysis)* |  | |  |
| Assumed risk estimate  *(e.g. baseline or population risk noted in Background)* |  | |  |
| Power *(e.g. power & sample size calculation, level of power achieved)* |  | |  |
| Notes: | | | |

# Data and analysis

*Copy and paste the appropriate table for each outcome, including additional tables for each time point and subgroup as required.*

***For randomised or non-randomised trial***

***Dichotomous outcome***

|  | Description as stated in report/paper | | | | | Location in text or source *(pg & ¶/fig/table/other)* |
| --- | --- | --- | --- | --- | --- | --- |
| Comparison |  | | | | |  |
| Outcome |  | | | | |  |
| Subgroup |  | | | | |  |
| Time point *(specify from start or end of intervention)* |  | | | | |  |
| Results | Intervention | | | Comparison | |  |
|  | No. with event | Total participants in group | | No. with event | Total participants in group |  |
|  |  |  |  |  |  |  |
| Any other results reported *(e.g. odds ratio, risk difference, CI or P value)* |  | | | | |  |
| No. missing participants |  | | |  | |  |
| Reasons missing |  | | |  | |  |
| No. participants moved from other group |  | | |  | |  |
| Reasons moved |  | | |  | |  |
| Unit of analysis *(by individuals, healthcare practitioner, clinic, hospital, community)* |  | | | | |  |
| Statistical methods used and appropriateness of these *(e.g. adjustment for correlation)* |  | | | | |  |
| Reanalysis required? *(If yes, specify why, e.g. correlation adjustment)* | Yes No Unclear | |  | | |  |
| Reanalysis possible? | Yes No Unclear | |  | | |  |
| Reanalysed results |  | | | | |  |
| Notes: | | | | | | |

***For randomised or non-randomised trial***

***Continuous outcome***

|  | | Description as stated in report/paper | | | | | Location in text or source *(pg & ¶/fig/table/other)* | |
| --- | --- | --- | --- | --- | --- | --- | --- | --- |
| Comparison | |  | | | | |  | |
| Outcome | |  | | | | |  | |
| Subgroup | |  | | | | |  | |
| Time point *(specify whether from start or end of intervention)* | |  | | | | |  | |
| Post-intervention or change from baseline? | |  | | | | |  | |
| Results | Intervention | | | Comparison | | |  | |
|  | Mean | SD *(or other variance, specify)* | No. participants | Mean | SD *(or other variance, specify)* | No. participants |  |  |
|  |  |  |  |  |  |  |  |  |
| Any other results reported *(e.g. mean difference, CI, P value)* | |  | | | | |  | |
| No. missing participants | |  | |  | | |  |  |
| Reasons missing | |  | |  | | |  |  |
| No. participants moved from another group | |  | |  | | |  |  |
| Reasons moved | |  | |  | | |  |  |
| Unit of analysis  *(by individuals, healthcare practitioner, clinic, hospital, community)* | |  | | | | |  | |
| Statistical methods used and appropriateness of these *(e.g. adjustment for correlation)* | |  | | | | |  | |
| Reanalysis required? *(specify)* | | Yes No Unclear | |  | | |  | |
| Reanalysis possible? | | Yes No Unclear | |  | | |  | |
| Reanalysed results | |  | | | | |  | |
| Notes: | | | | | | | | |

***For randomised or non-randomised trial***

***Other outcome***

|  | Description as stated in report/paper | | | | | Location in text or source *(pg & ¶/fig/table/other)* |
| --- | --- | --- | --- | --- | --- | --- |
| Comparison |  | | | | |  |
| Outcome |  | | | | |  |
| Subgroup |  | | | | |  |
| Time point *(specify from start or end of intervention)* |  | | | | |  |
| No. participant | Intervention | | | Control | |  |
|  |  |  |  |  |  |  |
| Results | Intervention result | SE (or other variance) | | Control result | SE (or other variance) |  |
|  |  |  |  |  |  |  |
|  | Overall results | | | SE (or other variance) | |  |
|  |  |  |  |  |  |  |
| Any other results reported |  | | | | |  |
| No. missing participants |  | | |  | |  |
| Reasons missing |  | | |  | |  |
| No. participants moved from other group |  | | |  | |  |
| Reasons moved |  | | |  | |  |
| Unit of analysis *(by individuals, healthcare practitioner, clinic, hospital, community)* |  | | | | |  |
| Statistical methods used and appropriateness of these |  | | | | |  |
| Reanalysis required? *(specify)* | Yes No Unclear | |  | | |  |
| Reanalysis possible? | Yes No Unclear | |  | | |  |
| Reanalysed results |  | | | | |  |
| Notes: | | | | | | |

***For Controlled Before-and-After study (CBA)***

|  | Description as stated in report/paper | | | | | Location in text or source *(pg & ¶/fig/table/other)* |
| --- | --- | --- | --- | --- | --- | --- |
| Comparison |  | | | | |  |
| Outcome |  | | | | |  |
| Subgroup |  | | | | |  |
| Time point *(specify whether from start or end of intervention)* |  | | | | |  |
| Post-intervention or change from baseline? |  | | | | |  |
| No. participants | Intervention | | | Control | |  |
|  |  |  |  |  |  |  |
| Results | Intervention result | SE *(or other variance, specify)* | | Control result | SE *(or other variance, specify)* |  |
|  |  |  |  |  |  |  |
|  | Overall results | | | SE *(or other variance, specify)* | |  |
|  |  |  |  |  |  |  |
| Any other results reported |  | | | | |  |
| No. missing participants |  | | |  | |  |
| Reasons missing |  | | |  | |  |
| No. participants moved from other group |  | | |  | |  |
| Reasons moved |  | | |  | |  |
| Unit of analysis *(by individuals, healthcare practitioner, clinic, hospital, community)* |  | | | | |  |
| Statistical methods used and appropriateness of these |  | | | | |  |
| Reanalysis required? *(specify)* | Yes No Unclear | |  | | |  |
| Reanalysis possible? | Yes No Unclear | |  | | |  |
| Reanalysed results |  | | | | |  |
| Notes: | | | | | | |

***For Interrupted Time Series study (ITS) or repeated measures study***

|  | Description as stated in report/paper | | | | | | Location in text or source *(pg & ¶/fig/table/other)* |
| --- | --- | --- | --- | --- | --- | --- | --- |
| Comparison |  | | | | | |  |
| Outcome |  | | | | | |  |
| Subgroup |  | | | | | |  |
| Length of time points measured  *(e.g. days, months)* |  | | | | | |  |
| Total period measured |  | | | | | |  |
| No. participants measured |  | | | | | |  |
| No. missing participants |  | | | | | |  |
| Reasons missing |  | | | | | |  |
|  | Pre-intervention | | | | Post-intervention | |  |
| No. time points measured |  | | | |  | |  |
| Mean value  *(with variance measure)* |  | | | |  | |  |
| Difference in means (post-pre) |  | | | | | |  |
| Percent relative change |  | | | | | |  |
| Any other results reported (with variance measure) |  | | | | | |  |
| Unit of analysis  *(by individuals, healthcare practitioner, clinic, hospital, community)* |  | | | | | |  |
| Statistical methods used and appropriateness of these |  | | | | | |  |
| Reanalysis required? *(specify)* | Yes No Unclear | | |  | | |  |
| Reanalysis possible? | Yes No Unclear | | |  | | |  |
| Individual time point results |  | | | | | |  |
| Read from figure? | Yes No |  | | | | |  |
| Reanalysed results | Change in level | | SE | | Change in slope | SE |  |
|  | |  | |  |  |
| Notes: | | | | | | | |

# For qualitative studies

|  | Description as stated in report/paper | | Location in text or source *(pg & ¶/fig/table/other)* |
| --- | --- | --- | --- |
| Outcome |  | |  |
| Subgroup (if applicable) |  | |  |
| **Time point** *(specify whether data was collected from start or end of intervention)* |  | |  |
| Length of time points  *(e.g. days, months)* |  | |  |
| No. participants |  | |  |
| No. missing participants |  | |  |
| Reasons missing |  | |  |
| Main themes/finding *(specify key quotes)* |  | |  |
| Sub-themes/finding *(specify key quotes)* |  | |  |
| Summary of qualitative finding |  | |  |
| Any other relevant results reported |  | |  |
| Type of analysis method used and its appropriateness |  | |  |
| Reanalysis required? *(specify)* | Yes No Unclear |  |  |
| Reanalysis possible? | Yes No Unclear |  |  |
| Notes: | | | |

# Other information

|  | **Description as stated in report/paper** | **Location in text or source** *(pg & ¶/fig/table/other)* |
| --- | --- | --- |
| **Key conclusions of study authors** |  |  |
| **Limitation of the study** |  |  |
| **Strategies to overcome the limitation** |  |  |
| **Recommendations** |  |  |
| **References to other relevant studies** |  |  |
| **Correspondence required for further study information** *(from whom, what and when)* |  | |
| **Further study information** **requested** *(from whom, what and when)* |  | |
| **Correspondence received** *(from whom, what and when)* |  | |
| **Notes:** | | |

.
